# Personality in the cockroach *Diploptera punctata*: Evidence for stability across developmental stages despite age effects on boldness

**DOI:** 10.1371/journal.pone.0176564

**Published:** 2017-05-10

**Authors:** Christina R. Stanley, Claudia Mettke-Hofmann, Richard F. Preziosi

**Affiliations:** 1 Faculty of Life Sciences, University of Manchester, Manchester, United Kingdom; 2 Department of Biological Sciences, University of Chester, Chester, United Kingdom; 3 School of Natural Sciences & Psychology, Liverpool John Moores University, Liverpool, United Kingdom; 4 School of Science and the Environment, Manchester Metropolitan University, Manchester, United Kingdom; University of Pretoria, SOUTH AFRICA

## Abstract

Despite a recent surge in the popularity of animal personality studies and their wide-ranging associations with various aspects of behavioural ecology, our understanding of the development of personality over ontogeny remains poorly understood. Stability over time is a central tenet of personality; ecological pressures experienced by an individual at different life stages may, however, vary considerably, which may have a significant effect on behavioural traits. Invertebrates often go through numerous discrete developmental stages and therefore provide a useful model for such research. Here we test for both differential consistency and age effects upon behavioural traits in the gregarious cockroach *Diploptera punctata* by testing the same behavioural traits in both juveniles and adults. In our sample, we find consistency in boldness, exploration and sociality within adults whilst only boldness was consistent in juveniles. Both boldness and exploration measures, representative of risk-taking behaviour, show significant consistency across discrete juvenile and adult stages. Age effects are, however, apparent in our data; juveniles are significantly bolder than adults, most likely due to differences in the ecological requirements of these life stages. Size also affects risk-taking behaviour since smaller adults are both bolder and more highly explorative. Whilst a behavioural syndrome linking boldness and exploration is evident in nymphs, this disappears by the adult stage, where links between other behavioural traits become apparent. Our results therefore indicate that differential consistency in personality can be maintained across life stages despite age effects on its magnitude, with links between some personality traits changing over ontogeny, demonstrating plasticity in behavioural syndromes.

## Introduction

The field of animal personality research has bloomed in recent years; inter-individual variation which was once considered background “noise” in behavioural ecological studies can now be formally attributed to among individual differences which persist through time [1]. Methods have recently been formalised and adapted [2] to show that personality (where individuals of the same species show consistent differences in their behaviour across time and contexts, [1, 3]) can be detected across a wide spectrum of animal species, including mammals [4, 5], birds [6, 7], fish [8, 9] and insects [10, 11]. The field has expanded to explore personality within a range of contexts such as mate choice [12], colour morphs [13, 14], collective movement [6, 15], dispersal [16], social network positions [17, 18], collective foraging [19] and leadership [20]. Suites of personality traits may also be correlated to form distinct behavioural syndromes [3]. However, whilst there is a wealth of published studies on animal personality to date, the development of behavioural consistency over ontogeny is an area which has often been neglected [21].

A central tenet of personality is its stability over time; however, significant physical and behavioural developmental changes are likely to occur over an individual’s lifetime and an understanding of how these changes affect behavioural traits is key to appreciating the adaptive value of personality itself. Experiences in early life can have significant influences upon the development of stable personality [22]. Periods of major reorganisation, such as morphogenesis, metamorphosis and sexual maturation, may also be expected to influence the stability of behaviour [21]. Juveniles often experience a very different set of selection pressures to adults, particularly where they live in completely different environments; in this context, behavioural stability is particularly surprising, especially when it occurs over complete metamorphosis (e.g. in the lake frog *Rana ridibunda*, [23], and the damselfly *Lestes congener*, [24]). Even in species where both adults and juveniles occupy similar environments, these life stages often have very different ecological needs, which affect their optimal behaviour; for example, most juvenile insects mainly focus on the search for food, whilst adults require mating partners or prime locations for oviposition or parturition [25]. Since personality traits may only become stable at the adult stage in some insect species (e.g. in the mustard leaf beetle *Phaedon cochleariae*, [26]) whilst these persist across life stages in others (e.g. *L*. *congener*, [24]), further studies are required to determine whether taxon or life history differences can best explain such differences in behavioural plasticity over ontogeny [26].

There may also be specific age and size effects on personality traits that involve risk-taking behaviour. In most insect species, juveniles and larvae are less mobile than adults and, due to their smaller size, they are at risk from a much wider range of predators; they therefore experience higher predation pressures, influencing both boldness and predator escape performance [27]. Their smaller size also imposes restrictions on the time they can spend without foraging, which may in turn promote bolder behaviour [26]; their greater metabolic requirements may be linked to a greater propensity to take risks [28, 29]. Life-history trade-offs may also occur [30]; the pace-of-life syndrome can explain links between behavioural traits and differences in either growth rates or physiology across life stages [31, 32] and explains why trade-offs between, for example, growth and mortality may differ across life stages [31]. These are all potential explanations for the finding that juvenile insects in some species have been shown to be bolder than their adult counterparts (e.g. field crickets *Gryllus integer*, [33]). The elucidation of mean-level changes in risk-taking behaviour across a broader range of species is now required to better understand the differential selection pressures in operation across life stages, and how these affect the development of personality [34].

To investigate behavioural consistency across discrete ontogenetic stages, an insect that undergoes a number of moults to adulthood is a perfect model. Despite the advantages of relatively short generation times, simple husbandry requirements and a vast variety of life history strategies, relatively few studies have assessed the consistency of personality across life stages in insects [10]. Indeed animal personality in general is frequently assessed over short time periods, or within a certain life stage, which is inadequate for the assessment of the proximate mechanisms contributing to personality variation [35]. In this study, consistency in individual behaviour will therefore be tested both within life stages (juvenile and adult) and across these stages in the gregarious cockroach *Diploptera punctata*, a species in which personality has not previously been explored despite its frequent use in endocrinological research [36].

A number of studies have so far examined personality variation in cockroaches; differential consistency in personality (where rank order in behaviour in a given context correlates across individuals over time, [21]) has so far been demonstrated in terms of exploration, sociality and foraging activity in male *Blattella germanica* [37], exploration, foraging, courtship, activity and boldness in *Gromphadorhina portentosa* [38–40] and sheltering behaviour in *Periplaneta americana* [41]. Influences of social isolation [37] and developmental environment [38] upon personality, characterisation of behavioural syndromes [39, 40] and collective personality at the group level [41] have also been explored. However, no studies have so far investigated changes in personality traits across life stages in cockroaches and all have so far focused upon males.

In order to investigate the development of behavioural consistency in *D*. *punctata*, we tested both nymphs and adults to explore 1) differential consistency in behavioural traits (both within and between life stages), 2) age effects on individual personality traits, 3) structural consistency (i.e. the extent to which correlations among behaviour patterns are preserved when measured in the same context(s) at a different time, [21]), 4) context generality (where scores across contexts correlate across individuals, [21]) in boldness within each life stage and 5) the effects of individual sex and size on behavioural traits.

Based on previous research and our understanding of the behavioural ecology of *D*. *punctata*, a number of factors may affect behavioural consistency in this species. Both juvenile and adult *D*. *punctata* inhabit a similar ecological niche [42]; consistency in personality traits across life stages may therefore be predicted, as was found in field crickets [33]. Age, however, may affect the magnitude of risk-taking behaviour comprising boldness and exploration; juveniles showed higher levels of boldness than adults in other insect studies [26, 33, 43]. Sex may affect boldness levels as this species shows distinct sexual size dimorphism (and hence differential predation risks), with females being significantly larger [36]. Sex effects upon both boldness [43, 44] and activity [26] have previously been demonstrated in other insects. Behavioural syndromes have also been identified in other cockroach species [39, 40]; if a behavioural syndrome is identified here, its stability over ontogeny will be investigated.

## Materials and methods

### Study population

Study individuals were taken from a mass colony of *D*. *punctata* maintained in laboratory conditions for over ten years. This colony was initially set up using individuals from three source populations and numbers have been maintained at a minimum population level of 200 individuals throughout this time, thus minimising the risk of inbreeding. These colonies were kept in an incubator at 24.5°c with a 12:12 light:dark cycle in plastic tanks approximately 33 x 26 x 19 cm, with ventilation provided in the lid. These cockroaches were allowed to feed *ad libitum* on Lidl’s “Orlando complete” dog biscuits and were given a constant supply of fresh water.

### Breeding and housing of focal individuals

Seventy-four nymphs were removed from their parents within 48 hours of hatching; these were from 17 different families (*X* + SD = 4.4 ± 2.0 nymphs per family). These nymphs were then housed separately from each other in one of three social environments, as part of another experiment; these were either in isolation, with a nymph companion or with an adult companion. Family and social environment were later considered in statistical analyses as factors potentially affecting behavioural consistency (see Statistical Analyses section). Housing consisted of transparent plastic containers of dimensions 11.5 x 11.5 x 6cm with air holes providing ventilation. Water was provided by Falcon tubes filled with water and plugged with soaked cotton wool. Water tubes were replaced as required. Nymphs were allowed to feed *ad libitum* on a 1:1 mixture of Aquarian fish flakes and Lidl’s “Orlando complete” dog biscuits.

All moults were recorded until the focal individuals reached adulthood (when wings are present). Upon reaching adulthood, individuals were photographed and their head width and pronotum width measured to the nearest 0.01cm using ImageJ 1.48 [45], with sex being determined by examination of the sexually dimorphic subgenital plates. Photographs were taken under standardized conditions; adults were placed in a petri dish on a white paper background with consistent background lighting. A ruler was placed next to the dish and included in the photograph to allow scale to be determined using the software. The accuracy of measurements was ascertained by ten adults being measured three times each and a repeatability analysis carried out in JMP (SAS Institute Inc., Cary, North Carolina). This gave a repeatability of 94.2% for head measurements and 97.4% for pronotum measurements. Since head and pronotum width were significantly correlated (Pearson’s correlation: *r*_*p*_ = 0.670, *N* = 60, *P* < 0.001), pronotum width was selected as the single measure of size due to its higher level of repeatability.

### Behavioural assays

Seventy-four individuals were tested in total; 24 were tested twice as third instar nymphs (10 males, 12 females, 2 unknown–died prior to reaching adulthood) and 65 as adults (29 males, 36 females) to explore differential consistency in behaviour within life stages. Since 19 individuals (7 males, 12 females) were tested both as juveniles and adults, these individuals alone were used to test for differential consistency of behavioural traits across life stages. The number of individuals tested at the third instar stage was limited by time constraints, mortality and inter-moult intervals, as has occurred in similar studies (e.g. [26]). Since the number of moults to adulthood varies between sexes and among individuals in *D*. *punctata* [36], we chose to test juveniles at the third instar stage since a minimum of three moults occurs in both sexes [36]. Individuals were not tested as fourth or fifth instars as only a small proportion go through these stages (usually females, [36]. A gap of *X* + SD = 32.8 + 10.8 days, *N* = 19 occurred between the second trial as a third instar and the first trial as an adult (lifespan of *D*. *punctata* is around 423 days, [42]). For both life stages, each individual was tested twice with a gap of three to seven days between testing so that differential consistency within each life stage could be established.

Three potential personality traits, boldness, exploration and sociality, were explored across three behavioural assays: the exploration arena, social arena and startle test. The order of testing was randomly assigned to each individual and its effects later considered (see Statistical Analyses section).

#### i. Exploration arena

**Fig 1:**
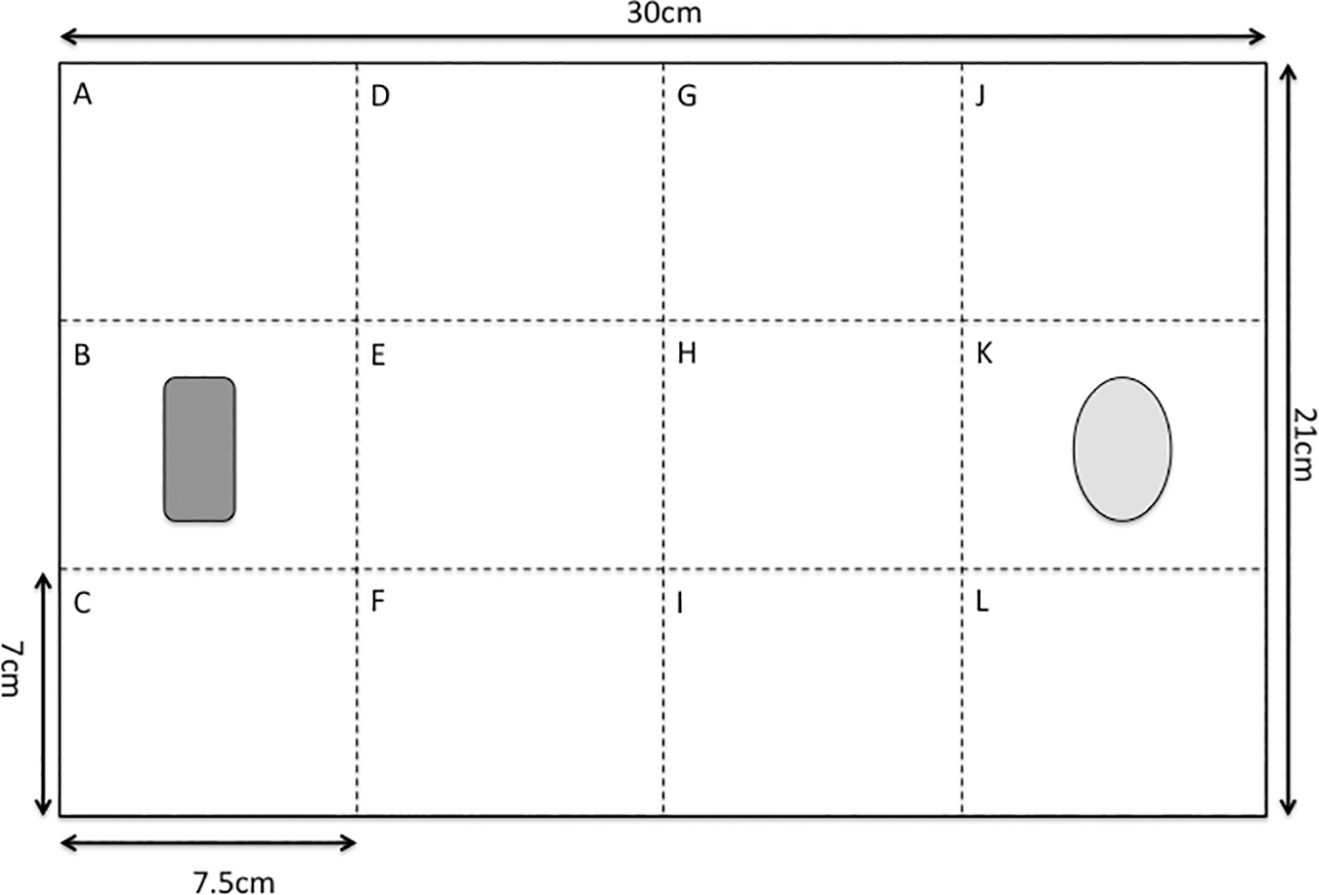
Diagram of testing arena. This illustrates the arena used in the exploration trial during behavioural testing in *Diploptera punctata*. A piece of A4 paper lined the bottom of the arena to mark the borders of sectors A to L; this was replaced for each new individual tested. Vaseline was applied to the sides of the arena above the paper to prevent cockroaches from climbing up the sides; the depth of the arena was approximately 8cm. The focal individual was introduced to the arena via an opaque tube placed in sector B. Sector K contained an empty plastic dish.

We used methods similar to those used for *B*. *germanica* [37]: a modified adaptation of the open field test (used to quantify exploration, [46]) with an emergence test component used as a measure of boldness [28]. The individual was removed from its normal housing and placed in an opaque perspex tube approximately 4cm in length and 3cm in diameter with both ends temporarily sealed (using petri dish lids as barriers) and left for three minutes to acclimatise. This tube was placed in sector B of an “exploration arena” prior to this acclimatisation time, with its temporarily sealed ends facing sectors A and C (Fig 1). The exploration arena was a 21x30cm plastic tray with a depth of 8cm. This contained a piece of A4 paper that divided the arena into 12 sectors (labelled A to L, Fig 1); these delimited distinct geographical areas of the arena e.g. corners, sides, and central portions. An empty oval-shaped plastic dish (dimensions approximately 4x3cm with a depth of 2cm) was placed in sector K. After the acclimatisation period, the barriers were removed and timing began.

We recorded when i) the head and ii) the entire body emerged from the tube and iii) when the focal individual crossed the centre line (separating sectors D-F and G-I). We also recorded the number of novel sectors explored within ten minutes. When either all sectors had been explored or ten minutes had elapsed, the experiment was terminated. If all twelve sectors were explored, the time at which the focal individual entered the last novel sector was recorded. The paper grid was replaced between individuals since aggregation of pheromones, present in faeces, may influence cockroach movement [47, 48].

#### ii. Social arena

This arena was identical to the exploration arena (Fig 1) except the plastic dish was replaced by a cotton net bag containing three adults randomly selected from the colony population (but ensuring both sexes were represented). The bag measured approximately 10 x 8 cm when flat (with an expanded volume of approximately 100cm^3^) and allowed individuals to move around the restricted space; antennal contact with the focal individual was also possible. Following three minutes of acclimatisation for the focal individual in the Perspex tube, timing began when the barriers sealing the plastic tube were removed.

We recorded the time at which the focal individual first entered the sector containing conspecifics (latency to reach conspecifics), the latency to make antennal contact with conspecifics and subsequent times when the focal individual both left and entered this sector, allowing the total time spent in the sector containing conspecifics to be calculated. The experiment was terminated after ten minutes.

If, after five minutes, the individual had not left the tube (which occurred in 21% of trials), we moved it to sector H, on the border of sector K; we rotated the tube by 90 degrees to ensure the individual within the tube was now facing the conspecifics. This was carried out to allow less bold or explorative individuals the opportunity to show social behaviour; these individuals may otherwise not leave the tube at all during the experiment hence would be scored low in terms of sociality as an artefact of their reduced boldness levels. The initial five minutes when these individuals did not leave the tube were included in the latency to both reach conspecifics and make antennal contact.

#### iii. Startle test

Using a methodology similar to that used for *G*. *portentosa* [40], an alternative emergence test to assay boldness was used to quantify an individual’s reaction to sudden exposure to light. The focal individual was placed in a 9cm diameter petri dish with an opaque lid and allowed to acclimatise for three minutes. Timing began when the lid was suddenly removed, exposing the focal individual to bright light. The times at which the individual first moved its i) antennae, ii) head and iii) initiated locomotion were recorded.

Across all three assays, each behavioural measure recorded was assigned to a particular personality trait based upon results from previous personality work in cockroaches [37, 40], with boldness being measured across two contexts (Table 1).

**Table 1:** Personality trait measurement.

Personality	Context	Assay	Measure
** trait**			
	Emergence	Exploration arena	Latency for head to emerge from tube
			Latency for body to emerge from tube
**Boldness**			
			Latency to move antennae
	Startle	Startle test	Latency to move head
			Latency to initiate locomotion
			Latency to cross centre line after emerging from tube
**Exploration**		Exploration arena	Number of sectors explored in ten minutes
			Time taken to explore all sectors
			Latency to reach conspecifics
**Sociality**		Social arena	Latency to touch antennae with conspecifics
			Total time spent with conspecifics

Measures used to assay each personality trait in *Diploptera punctata* across three behavioural assays.

### Statistical analyses

#### i. Differential consistency within and across life stages

In the exploration assay, 23 of 65 individuals never left their tubes in at least one trial; they were assigned a latency to leave the tube value of “601” if the event in question (head or body leaving tube, crossing centre line) never occurred. Since non-parametric correlations were later carried out, these individuals were therefore assigned the highest latency rank. In the social assay, again, 21 individuals never left their tubes at all in at least one trial, despite being moved after five minutes to within sight of the conspecifics. These were also assigned a value of 601 for each relevant latency (either to reach conspecifics or to touch antennae with conspecifics).

In order to test for differential consistency in each trait within each life stage, a composite measure was calculated for each separate personality trait to reduce the number of variables and hence enable a more powerful test. We collapsed the individual measures used for each personality trait into the first principal component (PC) score for each individual in the statistical package JMP, including scores from each trial for each individual. We then tested for a significant correlation between the two trials’ ranked PC scores for each individual by carrying out Spearman’s rank correlations in SPSS.

To test for differential consistency in personality traits across life stages, we again calculated PC scores for each personality trait for both life stages separately in the 19 individuals where these data were available; we calculated the mean latency for the two trials for each individual at each life stage then entered these means into the PC analysis. We again implemented a Spearman’s rank correlation to test for consistency within individuals. This combination of principle component analysis (PCA) and Spearman’s rank correlation tests was also used to show consistency in personality across metamorphosis in an anuran [23].

Since *D*. *punctata* are sexually dimorphic, sex differences in behaviour may occur. Males and females were therefore initially analysed separately, following the procedure outlined above. Further analyses were then carried out on pooled data where the direction of correlations was consistent between the sexes (S1 Appendix).

We repeated the correlations for the sociality assay excluding all individuals that were moved after five minutes of not leaving the tube to exclude the possibility that this practice was affecting results. Additionally, since the order of testing could affect the likelihood that individuals might leave the tube during the social assay (for example, if they had already experienced an experimental arena, this might affect their likelihood to leave the tube) and hence the measures used to assess sociality, a chi-squared test was carried out to examine whether order of testing had an effect on whether or not an individual left the tube during the social trial.

#### ii. Age effects

To test for age effects on the magnitude of individual behavioural measures between nymphs and adults, the difference between mean values obtained in trials at each life stage was calculated for each of the 19 individuals tested at both stages and a Wilcoxon signed-rank test was applied to test whether these differences significantly differed from zero. This allowed a non-parametric comparison between these repeats within individuals across life stages. Principal component scores were not used as the aim was to test for changes in the raw behavioural scores measured for each individual between life stages. Data were initially plotted for each sex separately to ensure there was not a consistent difference in the magnitude of the response between the sexes before these were pooled (S1 Appendix). If a difference was observed, each sex was analysed separately. This applied to three measures: latency to reach conspecifics, total time spent with conspecifics and total time taken to explore all sectors.

#### iii. Context generality & behavioural syndromes

Context generality in boldness was tested for by carrying out Spearman’s rank correlations between pairs of boldness measures taken in independent behavioural assays for both juveniles and adults. The context differed between assays since in the exploration arena, boldness was measured in terms of the latency for an individual to choose to leave a shelter, whereas in the startle test, boldness was measured in terms of the latency to move following sudden exposure to bright light by removal of the shelter.

Spearman’s correlations were carried out between thirteen pairs of individual measures quantifying different personality traits in different trials to test for the presence of behavioural syndromes in *D*. *punctata*. Pairings of measures quantifying different traits but collected in the same assay (e.g. exploration arena–latency for head to emerge from tube and latency to cross centre line) were excluded as they lacked independence. Since fewer measures were compared, the principal components approach was not necessary. Analyses were carried out for both nymphs and adults separately in order to determine whether any behavioural syndromes present persist across life stages (hence to test for structural consistency, [21]). Since the sociality measure “total time with conspecifics” could be dependent upon the latency to reach conspecifics (measured independently in the exploration assay), a lack of a correlation between these two measures could be used to justify these measures’ independence.

#### iv. Effects of sex, size, social environment & order of testing

To test for sex and size effects on adult personality, a mixed models approach was used to also incorporate potential effects of order of testing, social environment and family. A linear mixed-effects model was built for each adult personality dimension with its PC score as the response variable. A square root transformation was applied to normalise boldness PC scores prior to analysis, whilst exploration and sociality PCs were ranked for use in non-parametric analysis due to their non-conformity to any distribution. Standard data exploration procedures were carried out to ensure all data met the assumptions of the models [49]. Sex, order of testing, social environment and pronotum width were included as fixed factors, with family (brood) included as a random effect. Models were built in the R environment [50] using the nlme package [51]. Directionality of loading of each measure for each PC was used to interpret any significant effects.

All tests were two-tailed and the significance level was set at α = 0.05. *P*-values were corrected for multiple comparisons for each factor within each dataset by carrying out sequential Bonferroni corrections [52].

### Ethical note

We did not observe any adverse effects from the behavioural experiments conducted. The minimal number of individuals necessary to test the hypotheses was used and all animals were returned to the mass colony following final behavioural testing. Environmental enrichment (cardboard “egg boxes” to provide shelter and a more stimulating environment) was used in mass colonies, with small opaque plastic tubes being provided for developing nymphs as shelter.

## Results

### Differential consistency within and across life stages

For nymphs, the direction of most correlations between PC (first principal component) scores was consistent between the sexes (S1–S3 Figs) and so data were pooled for subsequent analyses. Since correlations appeared much weaker for females than males for nymph boldness and exploration (S1 Fig), and also for male boldness in adults (S2 Fig) and exploration between life stages (S3 Fig), these analyses were initially carried out separately for each sex. However, these analyses did not reveal significant sex differences (S1 Appendix), apart from the analysis of differential consistency in exploration between the life stages, which showed that this trend was likely to be driven by females (male: *r*_*s*_ = 0.321, *N* = 7, *P* = 0.482; female: *r*_*s*_ = 0.718, *N* = 12, *P* = 0.009). This result will be discussed further; however, pooling the sexes is still justified here due to the low power of this test to show a significant correlation for a sample of seven males.

Significant correlations between PC scores for both sexes pooled together provided evidence of differential consistency in boldness, exploration and sociality within adults, in boldness within third instars and in boldness and exploration across life stages (see Table 2 for PC loadings and the percentages of variance explained by this principal component, Table 3 for correlation test results, as well as S5–S7 Figs for individual correlation plots and S1 Table for means and SEs for all behavioural measures). These analyses could only be carried out using 63 of 65 adults due to missing data points for the social trial (see raw data in S2 Appendix).

**Table 2:** PCA loadings for personality trait consistency tests.

Life stage	Personality trait		Measure	Loading for PC1	% variance	Eigenvalue
					explained	
**3rd instar**	**Boldness**		Latency head emerges	0.758	63.5	3.18
(*N* = 24)			Latency body emerges	0.714		
			Latency move antennae	0.884		
			Latency move head	0.918		
			Latency initiate locomotion	0.684		
	**Exploration**		Latency to cross centre line	0.938	78	2.34
			No. sectors explored	-0.965		
			Total time taken	0.727		
	**Sociality**		Latency to reach conspecifics	0.665	47.6	1.43
			Latency to touch antennae with conspecifics	0.823		
** **	** **		Total time with conspecifics	-0.555		
**Adult**	**Boldness**		Latency head emerges	0.531	42.7	2.13
(*N* = 63)			Latency body emerges	0.586		
			Latency move antennae	0.714		
			Latency move head	0.682		
			Latency initiate locomotion	0.73		
	**Exploration**		Latency to cross centre line	0.943	80.5	2.41
			No. sectors explored	-0.957		
			Total time taken	0.78		
	**Sociality**		Latency to reach conspecifics	0.791	63.5	1.91
			Latency to touch antennae with conspecifics	0.774		
** **	** **		Total time with conspecifics	-0.825		

PCA loadings of measures used to generate first principal component scores (PC1) to assess consistency of strengths of personality traits within individuals within and between trials at each life stage in *Diploptera punctata*. For comparisons within life stages, all trials for all individuals were pooled prior to PCA. For comparisons across life stages, the mean value from the two trials carried out for each individual was calculated and these means were pooled prior to PCA. Sample sizes are given in parentheses.

**Table 3:** Tests for differential consistency in behavioural traits.

	Within		Within		Between	
	3rd instar		Adult		3rd instar & adult	
Trait	*r*_*s*_	*P*	*r*_*s*_	*P*	*r*_*s*_	*P*
						
**Boldness**	**0.469**	**0.02**	**0.61**	**<0.001**	**0.528**	**0.02**
**Exploration**	0.26	0.22	**0.442**	**<0.001**	**0.549**	**0.014**
**Sociality**	-0.074	0.732	**0.426**	**<0.001**	0.114	0.642

Summary of Spearman’s correlations between principal component scores (used as a composite measure for each behavioural trait, comprising multiple behavioural measures) to test for consistency in behavioural traits in *Diploptera punctata* both within each life stage (third instar and adult, comparing ranked scores from repeated trials) and between these life stages (comparing ranked mean scores from trials carried out at both nymph and adult stages). *P*-values remaining significant following a sequential Bonferroni test are shown in bold. Sample sizes are 24 third instars (10 male, 12 female, 2 unknown), 63 adults (28 male, 35 female) and 19 individuals measured across both life stages (7 male, 12 female).

After excluding all individuals that did not leave the tube during the sociality test in the first five minutes, results for a new correlation test between trials to examine differential consistency in sociality (Spearman’s rank correlations, third instars: *r*_*s*_ = -0.236, *N* = 10, *P* = 0.511; adults: *r*_*s*_ = 0.392, *N* = 38, *P* = 0.015; across life stages: *r*_*s*_ = -0.182, *N* = 11, *P* = 0.593) were relatively consistent with those obtained using all data; although the trend direction reversed for the between life stages analysis, the correlation remained extremely weak and non-significant. We can therefore conclude that the practice of moving these tubes did not significantly influence results. The order of testing (i.e. the order of behavioural assays carried out) did not affect the likelihood of individuals leaving the tube during the sociality test (Chi-square test: χ^2^_5_ = 6.02, *P* > 0.05), thus showing that previous experience of other trials did not affect the likelihood to leave the tube and hence the time spent with conspecifics in this social trial.

### Age effects

Plotting the data for each sex separately (S4 Fig) showed that the direction of change in magnitude of behavioural measures differed between the sexes for two sociality measures (latency to reach conspecifics and total time spent with conspecifics) and for one exploration measure (total time taken to explore all sectors). These measures were therefore initially analysed for males and females separately. However, following Bonferroni corrections, none of these sex specific differences were significant (S1 Appendix) and so both sexes were pooled for all subsequent analysis of all measures.

**Fig 2:**
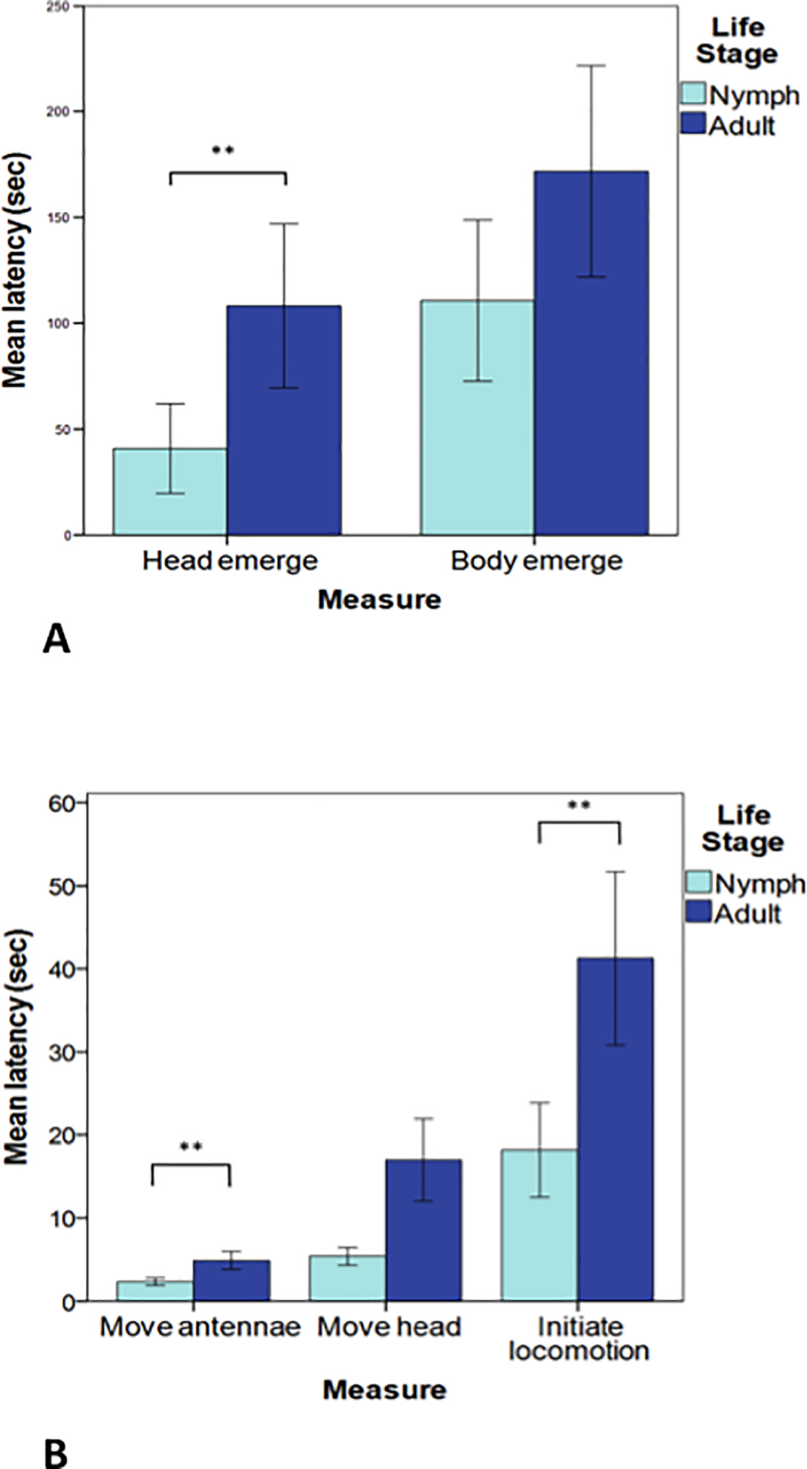
Age effects on boldness. *Diploptera punctata* individuals were significantly bolder as nymphs than as adults in terms of showing **A.** a shorter latency for their head to emerge from the tube in the exploration test, and **B.** a shorter latency to both move antennae and initiate locomotion in the startle test as nymphs. Error bars represent standard errors. Significant differences are marked by ** (*P* < 0.01). 19 individuals were tested across both life stages (7 males, 12 females).

Adults were significantly less bold than juveniles across three of the five measures of boldness tested (Fig 2). There was no apparent difference in levels of either exploration or sociality between the two life stages (Table 4).

**Table 4:** Summary of age effects tests.

Personality trait	Behavioural	Measure	*z*	*P*
	assay			
**Boldness**	Exploration	Latency head emerges	**179**	**0.003**
		Latency body emerges	99	0.121
	Startle	Latency move antennae	**128**	**0.002**
		Latency move head	119	0.023
		Latency initiate locomotion	**174**	**0.005**
**Exploration**	Exploration	Latency to cross centre line	22	0.735
		No. sectors explored	-17.5	0.725
		Total time taken	-40.5	0.497
**Sociality**	Social	Latency to reach conspecifics	45.5	0.445
		Latency to touch antennae with conspecifics	81	0.017
		Total time with conspecifics	-43.5	0.465

Results from Wilcoxon signed-rank test for age effects on personality measures within individuals, between third instar and adult life stages (*N =* 19: 7 male & 12 female) in *Diploptera punctata*. Results remaining significant following a sequential Bonferroni test are shown in bold.

### Context generality & behavioural syndromes

Consistent levels of boldness were evident in both juveniles and adults across the exploration and startle contexts from all measures used (Table 5), thus demonstrating context generality for this trait.

**Table 5:** Contextual consistency in boldness.

Context	Exploration	Startle	*r*_*s*_	*P*
** Measures**	Latency head emerges	Latency to move antennae	**0.631**	**0.001**
** correlated**		Latency move head	**0.683**	**<0.001**
**(3**^**rd**^ **instars)**		Latency initiate locomotion	**0.551**	**0.005**
	Latency body emerges	Latency to move antennae	**0.62**	**0.001**
		Latency move head	**0.628**	**0.001**
		Latency initiate locomotion	**0.524**	**0.009**
** Measures**	Latency head emerges	Latency to move antennae	**0.428**	**<0.001**
** correlated**		Latency move head	**0.351**	**0.004**
**(adults)**		Latency initiate locomotion	**0.303**	**0.014**
	Latency body emerges	Latency to move antennae	**0.394**	**0.002**
		Latency move head	**0.391**	**0.002**
		Latency initiate locomotion	**0.294**	**0.018**

Evidence for consistency in boldness levels across contexts in both third instars *(N* = 24: 10 male, 12 female & 2 unknown) and adults (*N* = 63: 28 male & 35 female) in *Diploptera punctata* was provided by significant two-tailed Spearman’s correlations between all combinations of independent measures. All *P*-values remained significant following a sequential Bonferroni test.

Within nymphs, exploration and boldness were found to significantly correlate across three pairs of measures (Table 6). No significant correlations were found between other pairs of measures. Within adults, there were significant correlations between sociality and both exploration (two pairs of measures) and boldness (two pairs of measures, Table 6). However, there was no evidence for a behavioural syndrome linking boldness and exploration in adults. There was therefore no evidence of structural consistency across life stages.

**Table 6:** Summary of tests for behavioural syndromes.

Traits	Measure1	Measure 2	Nymphs		Adults	
			*r*_*s*_	*P*	*r*_*s*_	*P*
**Exploration & Boldness**	Latency to cross	Latency move antennae	0.396	0.056	0.027	0.834
	centre line	Latency move head	0.519	0.009	0.036	0.778
		Latency initiate locomotion	**0.569**	**0.004**	0.321	0.009
	No. sectors	Latency move antennae	-0.493	0.014	-0.198	0.113
	Explored	Latency move head	**-0.646**	**0.001**	-0.138	0.272
		Latency initiate locomotion	**-0.666**	**<0.001**	-0.242	0.052
**Social & Exploration**	Total time with	Latency to cross centre line	0.037	0.862	**-0.485**	**<0.001**
	Conspecifics	No. sectors explored	0.06	0.781	**0.439**	**<0.001**
**Social & Boldness**	Total time with	Latency head emerges	0.145	0.500	**-0.404**	**0.001**
	Conspecifics	Latency body emerges	0.026	0.902	**-0.405**	**0.001**
		Latency move antennae	0.36	0.084	-0.173	0.169
		Latency move head	0.246	0.247	-0.007	0.955
		Latency initiate locomotion	0.119	0.578	-0.242	0.052

Results from Pearson’s correlations to test for the presence of behavioural syndromes in both nymphs and adults in *Diploptera punctata*. Correlations remaining significant following a sequential Bonferroni test are indicated by bold text. 63 adults and 24 nymphs were tested.

### Effects of sex, size, social environment & order of testing

**Fig 3:**
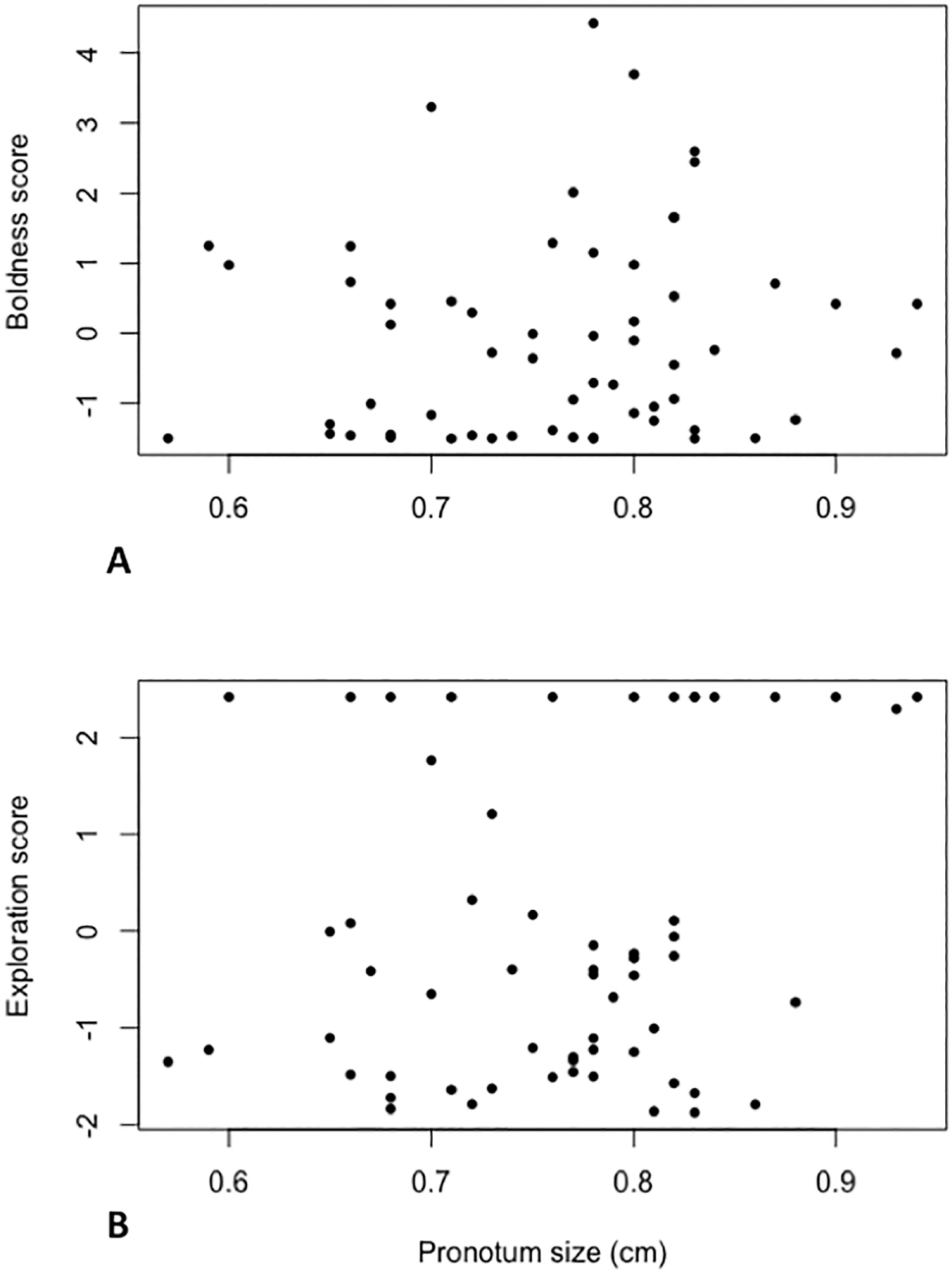
Summary of significant size effects results. Mixed models showed a significant effect of size on boldness and exploration in *Diploptera punctata*; larger individuals have higher PC scores (and therefore lower levels) of **A.** boldness and **B.** exploration. *N* = 60 adults.

Results from linear mixed effects models showed that neither social environment during rearing nor order of testing had significant effects on any of the three personality dimensions tested as adults (Table 7). Similarly, there was no apparent effect of sex on boldness or exploration. There was, however, a significant effect of adult size on both boldness and exploration, with larger individuals showing higher PC scores for both personality dimensions (Fig 3). Since a higher boldness PC score represented a greater latency to carry out all five behaviours measured for this trait, and a higher exploration PC score represented a greater latency to cross the centre line, fewer sections explored and a greater time taken to explore all sectors (Table 2), these results show that larger individuals are less bold and less explorative.

**Table 7:** Effects of size and sex.

Personality	Factor	Num DF	Den DF	*F*	*P*
dimension					
**Boldness**	(Intercept)	1	35	343.2	<0.001
	Sex	1	35	0.03	0.855
	Order	5	35	1.88	0.121
	Social environment	2	35	0.02	0.981
	Pronotum width	1	35	4.56	**0.04**
**Exploration**	(Intercept)	1	35	174.82	<0.001
	Sex	1	35	0.47	0.496
	Order	5	35	0.29	0.917
	Social environment	2	35	0.91	0.412
	Pronotum width	1	35	4.54	**0.04**
**Sociality**	(Intercept)	1	35	174.88	<0.001
	Sex	1	35	4.42	**0.043**
	Order	5	35	0.24	0.944
	Social environment	2	35	0.22	0.801
	Pronotum width	1	35	1.46	0.235

Linear mixed-effects models found a significant effect of size (measured by adult pronotum width) on both boldness and exploration scores and effects of sex on sociality scores in *Diploptera punctata*, as shown in bold. The order of testing (i.e. order trials were carried out) and social environment (isolated, with adult or with nymph companion) during development did not significantly affect these measures. Brood identity was included as a random factor. Degrees of freedom are presented for both denominator (Den DF) and numerator (Num DF). 60 individuals (for which all data were available) were included in the model.

**Fig 4:**
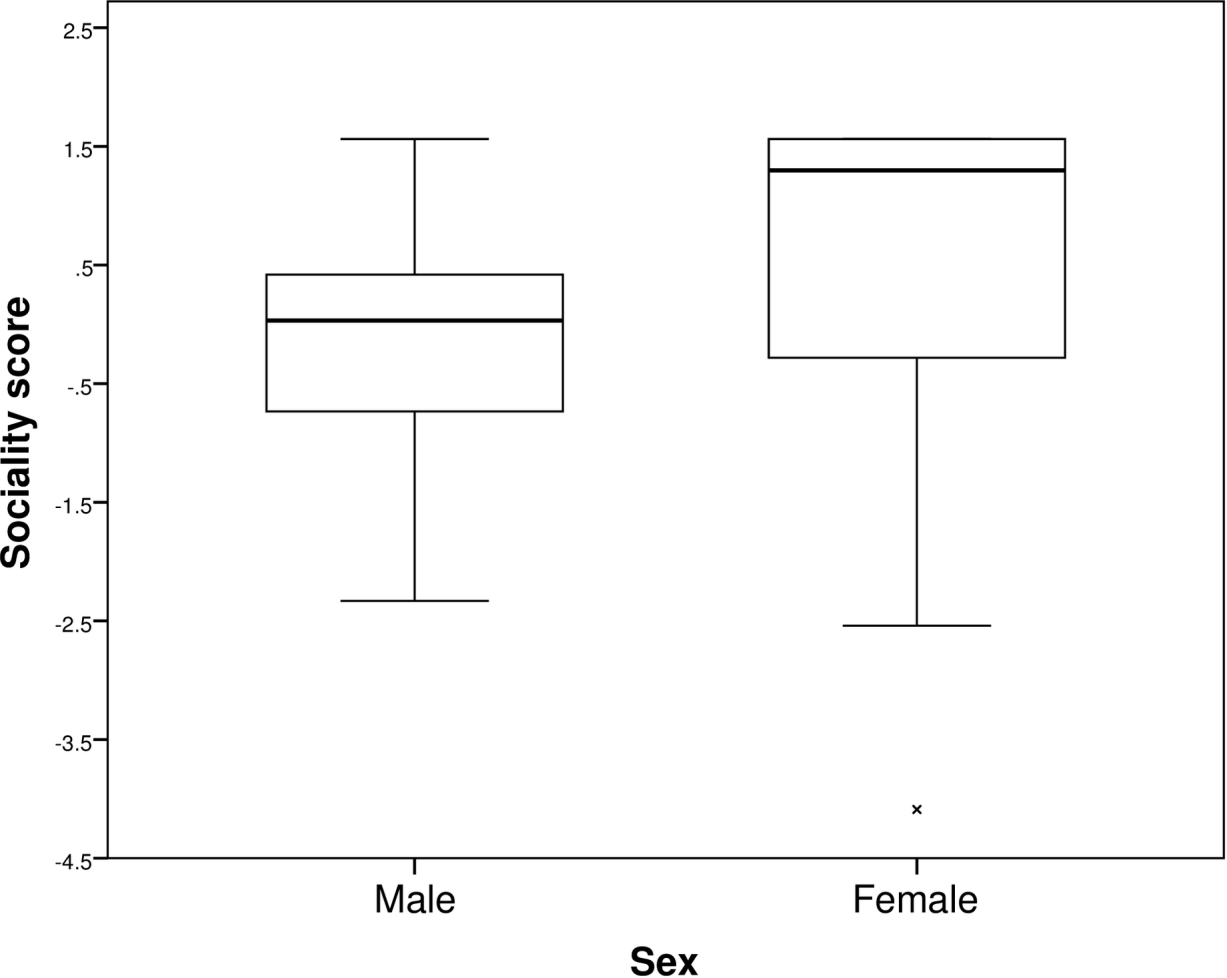
Summary of significant sex effects results. Mixed models showed a significant effect of sex on sociality in *Diploptera punctata*; males had lower sociality PC scores than females. *N* = 60 adults. The boxes are bounded by the upper and lower quartiles and are divided by the median. Maximum and minimum values within 1.5 box lengths of the quartiles are represented by the ends of whiskers and values outside of this range are shown by a cross.

There was an apparent effect of sex on sociality, with males having a lower PC score than females (Fig 4). Since a higher PC score represented a greater latency to reach and touch antennae with conspecifics and a shorter overall time spent with conspecifics (Table 2), these results indicate that males showed more motivation to approach conspecifics than did females.

## Discussion

Inter-individual variation in personality was evident in *D*. *punctata* cockroaches; whilst in third instars only boldness was shown to be consistent, in adults differential consistency was evident in boldness, exploration and sociality. Moreover, in the sample tested, we found boldness and exploration were stable across life stages despite age effects on population boldness levels. Therefore, boldness was the only personality trait to be consistent within and across all tested life stages, whereas exploration and sociality only emerged as stable in adults. Behavioural syndromes were found in both nymphs and adults but for different traits, indicating a lack of consistency across stages. We also found evidence of context generality in boldness within both juveniles and adults. There were clear effects of sex and size; larger individuals were less bold and less exploratory, whilst males showed higher levels of sociality than females.

### Differential consistency in behaviour within life stages

Our demonstration of differential consistency in boldness in *D*. *punctata* nymphs, as well as in boldness, exploration and sociality in adults (Table 3), clearly indicates the existence of personality in cockroaches. Our results are consistent with other studies in which boldness was found to be consistent in nymphs of field crickets [53] and damselflies *zygoptera* [24] but contrasts with results found in mustard leaf beetles [26]. The two former studies were carried out on a particular instar stage, as was our study, whereas the latter study on the beetles was carried out across several instar stages. Interestingly, only the studies conducted within a particular instar report consistency in boldness. Further research is needed to determine whether consistency can also be found across instar stages. Consistency in boldness in adults has previously been documented in a variety of insects such as seed beetles *Callosobruchus maculatus* [54], mustard leaf beetles [26], firebugs *Pyrrhocoris apterus* [43] and hissing cockroaches [40]. Exploration was also shown to be consistent in adult hissing cockroaches [38] and firebugs [43], whilst sociality was shown to be consistent in *B*. *germanica* [37] and courtship display behaviour was shown to be consistent in hissing cockroaches [39]. Since *D*. *punctata* has been widely used for physiological research [36], an appreciation of its inter-individual differences now places it as a prime candidate for the exploration of physiological changes correlated with personality trait variation.

The result that exploration and sociality were not found to be significantly consistent in third instar nymphs may indicate that these personality traits are not yet stable at this developmental stage. However, our demonstration of differential consistency in exploration across life stages is at odds with this explanation, at least for exploration behaviour, as this implies that exploration is already established at this life stage. Another potential explanation is that these personality traits may be more unstable during periods of rapid morphological change such as frequent moulting during juvenile development, which often require major reorganisation [21]. Indeed other studies on squid *Euprymna tasmanica* [55] and red junglefowl *Gallus gallus* [56] have failed to find consistency in various personality traits (boldness, tonic immobility, exploration and predator responses) within early developmental stages. It seems that specific traits are selected for consistency depending on the taxon but that other traits are inconsistent at an early developmental stage. Selection for greater behavioural plasticity may be beneficial at an early life stage, where future environments are less predictable [55, 56]. Further work with a larger sample of nymphs is required to better understand this result since it may also be explained by relatively low statistical power.

### Differential consistency in behaviour across life stages

Stability in personality across discrete life stages has previously been demonstrated in only a handful of species, for example in the damselfly *L*. *congener* [24], the firebug [43], the lake frog *R*. *ridibunda* [23] and the laboratory rat *Rattus norvegicus* [57]. In others, such as the mustard leaf beetle [26], personality levels were only stable at the adult stage, whilst individual consistencies were found to be generally low across the major developmental stages of becoming independent and sexually mature in the jungle fowl *G*. *gallus* [56].

Despite a general lower likelihood of repeatability in behavioural experiments in ectotherms compared to endotherms [58] we found exploration and boldness remained consistent across discrete life stages in our sample of cockroaches (Table 3), in line with our prediction. Nymphs and adults share similar environments, lifestyles and possibly foraging strategies [25]. Consistent individual strategies to collect information (exploration) and to respond to risky situations (boldness) may therefore be adaptive across developmental stages. As a consequence, boldness and exploration seem to become “fixed” at the juvenile stage in *D*. *punctata*. However, it should be noted that consistency in exploration was mainly driven by females. Whether males do not show consistency in exploration across life stages or whether this result is due to low statistical power resulting from a small sample size requires further research. In contrast, sociality was not consistent across life stages and may be shaped by factors only arising once a discrete life stage such as sexual maturity has occurred. The stabilisation of hormonal profiles which occurs at this point [56] may have an effect here as well. Social factors, such as societal roles, may also affect the stability of certain personality traits [59, 60].

### Age effects

As expected, juveniles showed consistently higher levels of boldness than adults in three measures across two contexts in our sample (Fig 2), which is consistent with other studies on insects [26, 33, 43]. Whilst nymphs and adults may inhabit the same environment and may have the same lifestyle and foraging habits, allowing them to use the same individual strategies (i.e. showing relative consistency between individuals [21]), they may be exposed to environmental challenges to different degrees resulting in variation in the magnitude of behaviours expressed [21]; examples are differences in predation risk or life-history trade-offs. According to the asset protection hypothesis [30] adults may be more cautious in unfamiliar situations so as not to miss opportunities for reproduction, whereas juveniles may take greater risks to reach the reproductive stage as quickly as possible; this hypothesis is supported by studies on field crickets [33, 44]. Another factor responsible for variation in environmental challenges is body size. Size effects on boldness have previously been demonstrated in mustard leaf beetles [26] and hissing cockroaches [38]. The higher boldness levels of smaller individuals were attributed to their greater metabolic requirements and therefore higher willingness to take risks. Whether life-history, body size or both factors are responsible for differences in boldness between nymphs and adults in our study species needs further investigation.

Surprisingly, exploration did not differ between age classes even though exploration has previously been found to be higher in juveniles than adults [14, 61–63] across different taxa. The formation of different behavioural syndromes within life stages in our study may play a role here. While exploration was positively correlated with boldness in nymphs, it was positively correlated with sociality in adults. Whether the correlation with other traits constrains exploration [64] or whether exploration is important in different contexts across life stages (e.g. finding food in nymphs versus finding food and mates as adults) is an exciting next step to investigate.

### Context generality & behavioural syndromes

We found evidence for context generality in boldness in both juvenile and adult *D*. *punctata* (Table 5). *C*ontext generality is not always demonstrable in boldness [8, 65], despite its inclusion in some definitions of personality [1]. Whilst boldness may be adaptive in certain situations, such as in intraspecific competition for resources, bold individuals may be at a disadvantage when confronted by a predator [66]. Personality may therefore affect individual fitness in context-dependent ways [67], which may explain why variation in personality persists [3]. In our study, the two contexts (latency to leave an opaque tube and latency to move following a sudden stimulus) may both be linked as common responses to an immediate threat from predation, which could explain their correlation. Consistency within contexts was also found for two boldness measures (time to leave tube after disturbance and time to walk after thrown into a novel arena) in firebugs [43]. In contrast, the two boldness measures (latency to leave cover after disturbance and latency to move after squeezing) tested in mustard leaf beetles were not correlated [26].

Within nymphs alone, we found significant relationships between exploration and boldness using three pairs of measurements across two independent pairs of trials. We therefore provide evidence of a behavioural syndrome in nymphs where boldness and exploration are linked (Table 6). Since these same correlations were not apparent within adults (despite a much larger sample size), it is likely that this behavioural syndrome is specific to nymphs. Since obtaining food to shorten the latency to reach a reproductive stage is an essential driver for nymphs [25], boldness and exploration may combine to improve the efficiency of foraging, whilst for adults, these traits diverge for other purposes.

In adults, we found evidence of a behavioural syndrome linking sociality (total time spent with conspecifics) with exploration (two measures), as well as one linking sociality (total time spent with conspecifics) with boldness (two measures; Table 6). It could be argued that these correlations are an artefact of our experimental design and not true behavioural syndromes as the total time spent with conspecifics is likely to be dependent on an individual’s boldness or exploration; if they are slow to leave their tube or cross the centre line, they will have less time available to spend with conspecifics. However, the lack of significant correlations between these pairs of measures in nymphs (despite boldness and exploration showing a significant correlation) provides evidence against the potential confounding effects of boldness or exploration on sociality in this assay. It is therefore likely that these behavioural syndromes are adaptive in adults, but not in nymphs, and this experimental design is therefore justified for exploring these three behavioural traits independently.

Since no behavioural syndrome was consistently found in both adults and nymphs by this experiment, we can provide no evidence of structural consistency [21] in personality in this species. There are currently very few studies that address the persistence of behavioural syndromes over ontogeny; however, those which do often fail to find evidence of structural consistency (e.g. [56, 64]). This is perhaps explained by the differential selection pressures that adults and juveniles are often exposed to; it may therefore be adaptive for the organisation of behaviours into syndromes to change over development [21, 68].

### Effects of sex and size

We found adult size (but not sex) significantly affected both boldness and exploration; larger individuals were both less bold and less explorative (Fig 3). This result contrasts with a similar study on *G*. *portentosa* which found no effect of size on risk-acceptance (which includes behaviours associated with boldness, such as exploration and food acquisition, [28]). However, smaller size in individuals which were kept in a low nutrition environment during this study was associated with increased risk-taking behaviour (in terms of exploration, foraging and recovery after disturbance, [38]). Size, therefore, only had an effect under stronger competitive conditions. A relationship between body size and boldness has also been found in fish such as the poeciliid *Brachyraphis episcopi* and the guppy *Poecilia reticulata* [28, 29]. Smaller individuals may have greater metabolic requirements and are therefore more willing to take risks [28, 29]. This could also be the case in *D*. *punctata*. The higher exploration levels in smaller individuals may be explained by subordination as larger individuals may monopolise resources requiring smaller individuals to invest more in exploration for uncontested resources.

Sex did not significantly affect either boldness or exploration in adults, despite the high levels of sexual dimorphism in this species [36], but males were found to be more motivated to approach conspecifics than were females, as demonstrated by males’ lower sociality scores (Fig 4). This result is likely to reflect sex differential reproductive motivation in *D*. *punctata*, although there is little known regarding mating behaviour and sexual selection in this species. Where sex differences in personality are apparent, they are likely to be explained by differential selection on male and female personality, perhaps explained by intrasexual selection, mate choice, differential reproductive roles, ecological demands or life histories [69]. In this case, males may be more motivated to approach conspecifics in order to obtain matings than are females, whose behaviour is often adapted to minimise the costs of male coercion [70]. The lack of sex differences in both exploration and boldness is unexpected; perhaps sex-differential selection upon personality is low in cockroaches as pressures such as predation act equally on both males and females. Indeed in species such as field crickets where there are clear sex differences in predation pressure (due to male crickets’ calls attracting the attention of predators), sex differences have been found in differential consistency across life stages [44].

## Conclusions and future work

Here we show evidence of differential consistency in personality both within and across life stages in cockroaches, as well as age effects upon boldness and a lack of stability in a behavioural syndrome over development in the sample tested. We show that differential consistency can be maintained despite age effects on the magnitude of personality traits, as well as showing that there is flexibility in the linkage between behavioural traits at different life stages.

Further work could reveal whether consistent behavioural variation is adaptive in the group context; testing individuals in isolation may not be a true representation of their personality in a group as behaviour may be modified by the influence of other group members [71] and isolated individuals may behave in a qualitatively different way to those in groups [72]. Personality sampling in wild populations may also provide crucial information on the many potential factors promoting personality variation [69], especially since this may have a significant impact upon survival in the natural habitat [53].

## Supporting information

S1 Fig Sex differential nymph behavioural trait correlations. Directions of correlations between PC1 scores across trials 1 and 2 (T1 and T2) for nymphs, separated by sex, for the three behavioural traits assayed (boldness in **a.** males and **b.** females, exploration in **c.** males and **d.** females, sociality in **e.** males and **f.** females). *N* = 22 (10 males, 12 females).
(TIF)

S2 Fig Sex differential adult behavioural trait correlations. Directions of correlations between PC1 scores across trials 1 and 2 (T1 and T2) for adults, separated by sex, for the three behavioural traits assayed (boldness in **a.** males and **b.** females, exploration in **c.** males and **d.** females, sociality in **e.** males and **f.** females). *N* = 63 (28 males, 35 females).
(TIF)

S3 Fig Sex differential behavioural trait correlations across life stages. Directions of correlations between PC1 scores for individuals across life stages, separated by sex, for the three behavioural traits assayed (boldness in **a.** males and **b.** females, exploration in **c.** males and **d.** females, sociality in **e.** males and **f.** females). *N* = 19 (7 males, 12 females).
(TIF)

S4 Fig Magnitude of age effects. Bar charts for **a.** males and **b.** females showing the mean and standard error change in each behavioural measure from nymph to adult life stages. Behavioural measures quantify boldness (latency for head, B1, and body, B2, to emerge; latency to move antennae, B3 and head, B4; latency to initiate locomotion, B5), exploration (latency to cross centre line, E1; no. sectors explored, E2; total time taken, E3) and sociality (latency to reach, S1, and touch, S2, conspecifics; total time with conspecifics, S3). *N* = 19 (7 males, 12 females).
(TIF)

S5 Fig Nymph differential consistency. Plots of Spearman’s rank correlations showing levels of differential consistency in nymph **a.** boldness, **b.** exploration and **c.** sociality. *N* = 24.
(TIF)

S6 Fig Adult differential consistency. Plots of Spearman’s rank correlations showing levels of differential consistency in adult **a.** boldness, **b.** exploration and **c.** sociality. *N* = 63.
(TIF)

S7 Fig Differential consistency across life stages. Plots of Spearman’s rank correlations showing levels of differential consistency in **a.** boldness, **b.** exploration and **c.** sociality across life stages. *N* = 19.
(TIF)

S1 Table Behavioural measure descriptive statistics. Mean and standard error for each behavioural measure for each life stage (“combined mean”), with means and standard errors also presented separately for males and females. All values are in seconds except for the number of sectors explored. Sample sizes are 22 3^rd^ instars (10 male, 12 female) and 63 adults (28 male, 35 female).
(DOCX)

S1 Appendix Sex-specific differential consistency analyses and sex-specific age effects analyses. (DOCX)

S2 Appendix Complete dataset containing all raw data used for personality analyses on *Diploptera punctata*. (XLSX)
