# Supplementary figures and images for: Personality in the cockroach Diploptera punctata: Evidence for stability across developmental stages despite age effects on boldness
Source: PLoS One. 2017 May 10;12(5):e0176564. doi: 10.1371/journal.pone.0176564 (PMC5425029; doi:10.1371/journal.pone.0176564)

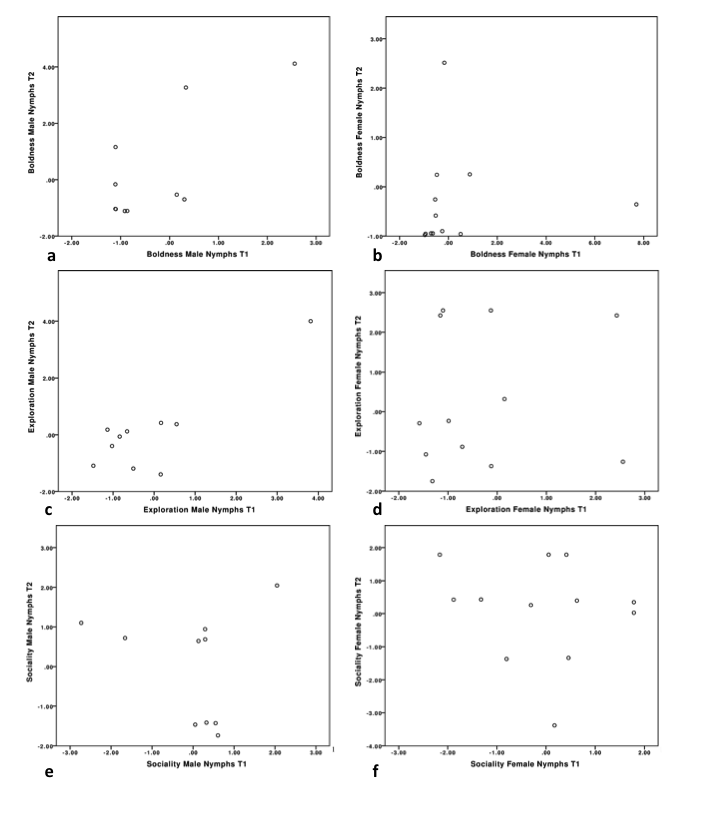

Supplement: S1 Fig — Directions of correlations between PC1 scores across trials 1 and 2 (T1 and T2) for nymphs, separated by sex, for the three behavioural traits assayed (boldness in a. males and b. females, exploration in c. males and d. females, sociality in e. males and f. females). N = 22 (10 males, 12 females). (TIF) [file pone.0176564.s001.tif]

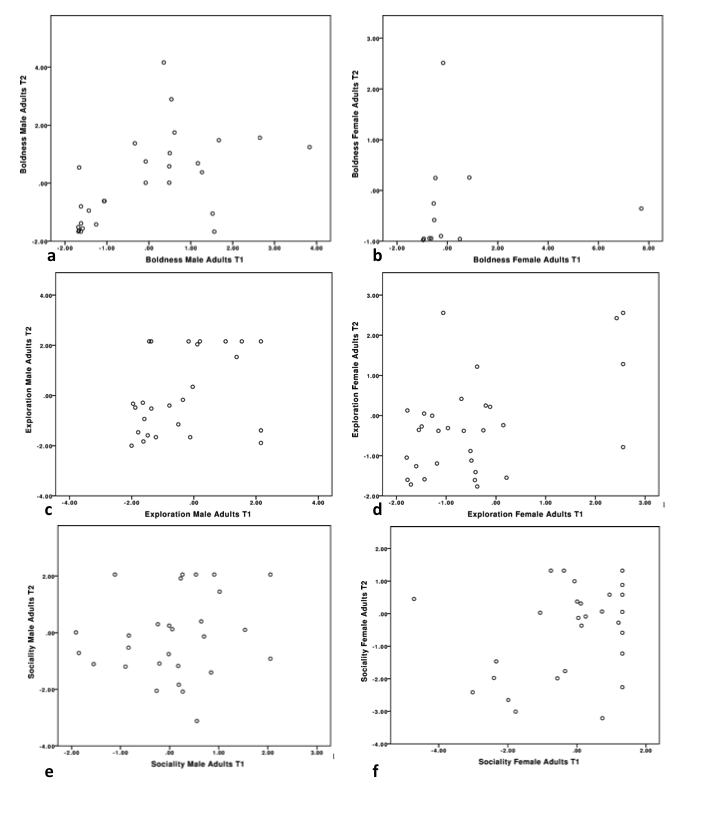

Supplement: S2 Fig — Directions of correlations between PC1 scores across trials 1 and 2 (T1 and T2) for adults, separated by sex, for the three behavioural traits assayed (boldness in a. males and b. females, exploration in c. males and d. females, sociality in e. males and f. females). N = 63 (28 males, 35 females). (TIF) [file pone.0176564.s002.tif]

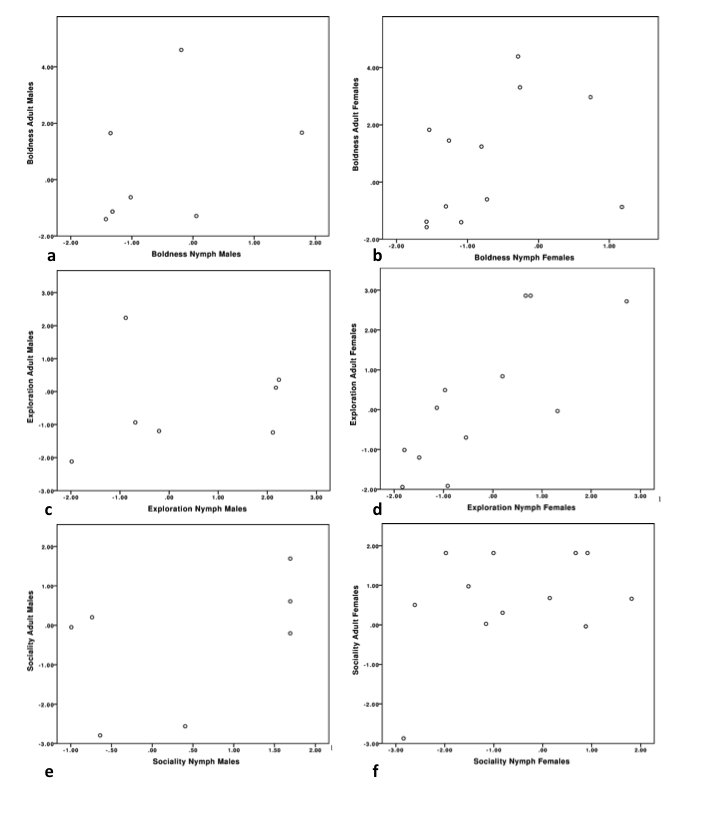

Supplement: S3 Fig — Directions of correlations between PC1 scores for individuals across life stages, separated by sex, for the three behavioural traits assayed (boldness in a. males and b. females, exploration in c. males and d. females, sociality in e. males and f. females). N = 19 (7 males, 12 females). (TIF) [file pone.0176564.s003.tif]

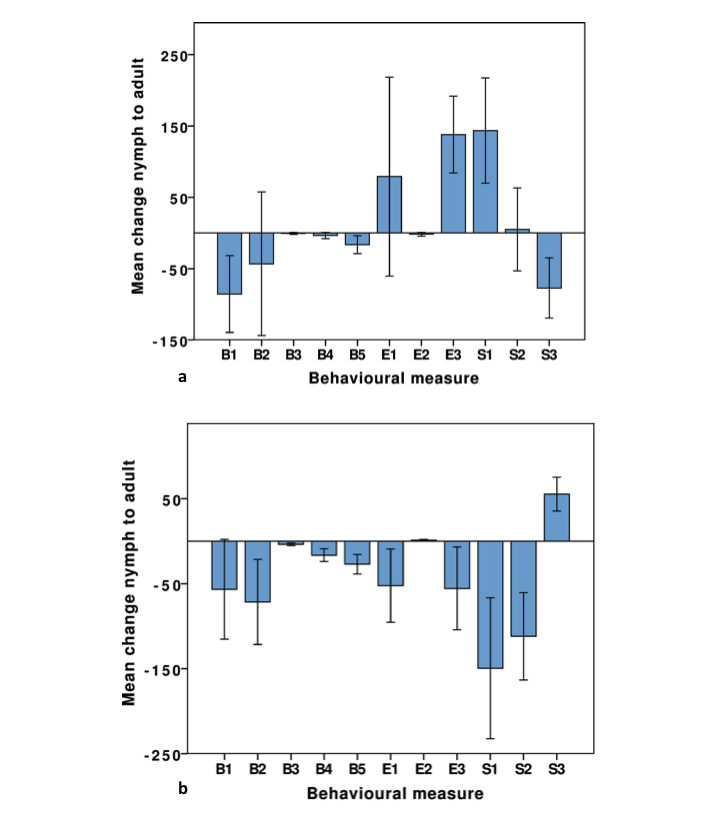

Supplement: S4 Fig — Bar charts for a. males and b. females showing the mean and standard error change in each behavioural measure from nymph to adult life stages. Behavioural measures quantify boldness (latency for head, B1, and body, B2, to emerge; latency to move antennae, B3 and head, B4; latency to initiate locomotion, B5), exploration (latency to cross centre line, E1; no. sectors explored, E2; total time taken, E3) and sociality (latency to reach, S1, and touch, S2, conspecifics; total time with conspecifics, S3). N = 19 (7 males, 12 females). (TIF) [file pone.0176564.s004.tif]

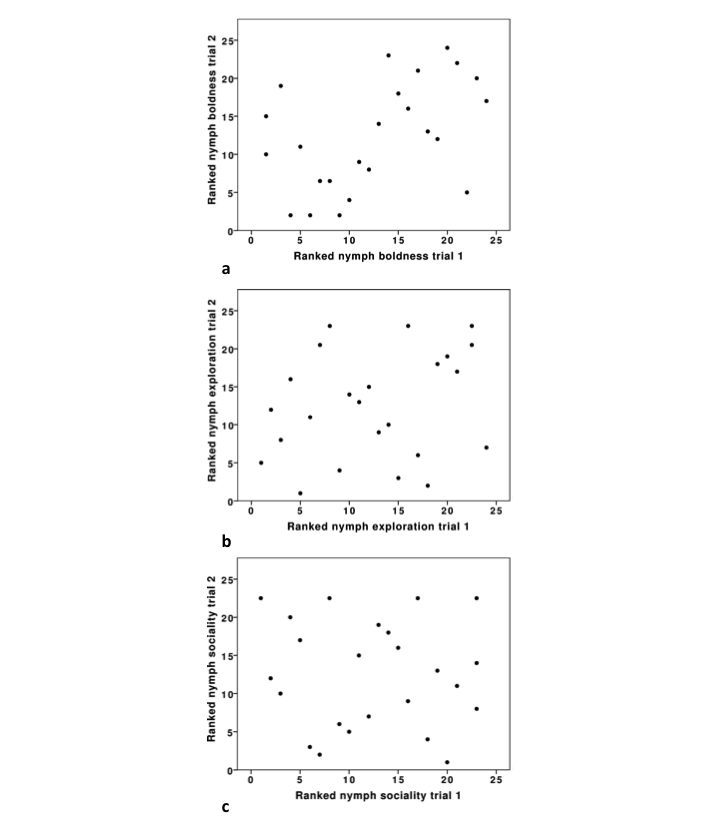

Supplement: S5 Fig — Plots of Spearman’s rank correlations showing levels of differential consistency in nymph a. boldness, b. exploration and c. sociality. N = 24. (TIF) [file pone.0176564.s005.tif]

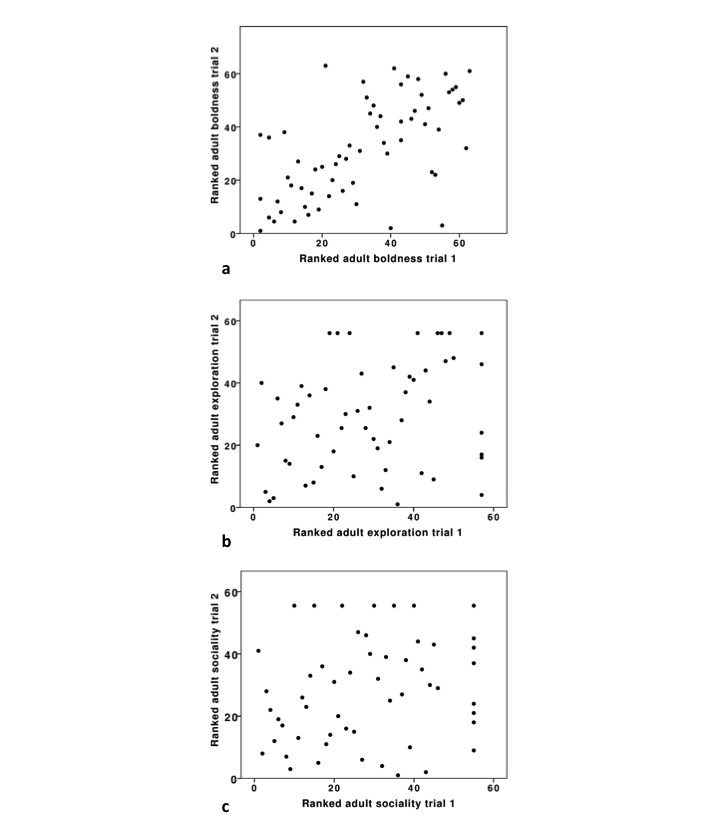

Supplement: S6 Fig — Plots of Spearman’s rank correlations showing levels of differential consistency in adult a. boldness, b. exploration and c. sociality. N = 63. (TIF) [file pone.0176564.s006.tif]

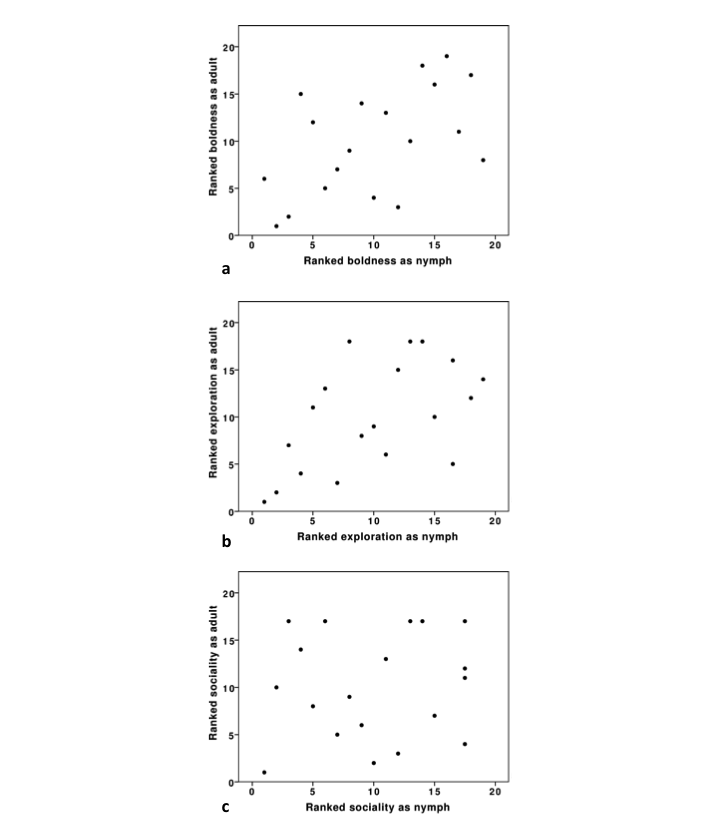

Supplement: S7 Fig — Plots of Spearman’s rank correlations showing levels of differential consistency in a. boldness, b. exploration and c. sociality across life stages. N = 19. (TIF) [file pone.0176564.s007.tif]
