# Supplementary material for: Personality in the cockroach Diploptera punctata: Evidence for stability across developmental stages despite age effects on boldness
Source: PLoS One. 2017 May 10;12(5):e0176564. doi: 10.1371/journal.pone.0176564 (PMC5425029; doi:10.1371/journal.pone.0176564)
Supplement: S1 Table — Mean and standard error for each behavioural measure for each life stage (“combined mean”), with means and standard errors also presented separately for males and females. All values are in seconds except for the number of sectors explored. Sample sizes are 22 3rd instars (10 male, 12 female) and 63 adults (28 male, 35 female). (DOCX) [file pone.0176564.s008.docx]

| **Life stage** | **Personality** | **Measure** | **Male** | **Female** | **Combined** |
| --- | --- | --- | --- | --- | --- |
|  | **dimension** |  | **Mean (SE)** | **Mean (SE)** | **Mean (SE)** |
|  |  |  |  |  |  |
| **3rd instar** | **Boldness** | Latency head emerges | 39.6 (32.8) | 55.7 (29.8) | 43.1 (18.7) |
|  |  | Latency body emerges | 46.8 (32.7) | 114.6 (39.0) | 75.3 (23.5) |
|  |  | Latency move antennae | 4.4 (1.9) | 6.2 (3.6) | 4.9 (1.9) |
|  |  | Latency move head | 6.4 (2.2) | 13.0 (6.4) | 9.0 (3.3) |
|  |  | Latency initiate locomotion | 15.3 (6.0) | 33.4 (9.6) | 22.6 (5.6) |
|  |  |  |  |  |  |
|  | **Exploration** | Latency to cross centre line | 95.7 (63.9) | 193.0 (53.0) | 136.0 (36.2) |
|  |  | No. sectors explored | 9.7 (1.2) | 8.8 (1.1) | 9.5 (0.7) |
|  |  | Total time taken | 430.8 (59.4) | 413.0 (44.0) | 407.2 (31.1) |
|  |  |  |  |  |  |
|  | **Sociality** | Latency to reach conspecifics | 150.6 (59.3) | 188.5 (43.7) | 163.7 (30.3) |
|  |  | Latency to touch conspecifics | 515.4 (29.3) | 471.8 (39.4) | 504.3 (23.1) |
|  |  | Total time with conspecifics | 173.5 (116.4) | 101.7 (23.1) | 122.1 (43.5) |
|  |  |  |  |  |  |
| **Adult** | **Boldness** | Latency head emerges | 175.8 (37.1) | 102.1 (32.5) | 133.7 (24.0) |
|  |  | Latency body emerges | 209.2 (41.6) | 148.9 (38.3) | 176.9 (27.7) |
|  |  | Latency move antennae | 3.8 (0.6) | 4.1 (0.7) | 4.0 (0.5) |
|  |  | Latency move head | 9.5 (1.6) | 16.0 (3.0) | 12.9 (1.8) |
|  |  | Latency initiate locomotion | 36.7 (7.1) | 44.6 (7.0) | 40.6 (4.9) |
|  |  |  |  |  |  |
|  | **Exploration** | Latency to cross centre line | 222.4 (41.7) | 182.8 (39.0) | 204.2 (28.2) |
|  |  | No. sectors explored | 7.9 (0.8) | 8.7 (0.8) | 8.4 (0.5) |
|  |  | Total time taken | 425.8 (30.3) | 435.9 (28.5) | 434.4 (20.1) |
|  |  |  |  |  |  |
|  | **Sociality** | Latency to reach conspecifics | 159.6 (30.4) | 321.9 (35.3) | 248.3 (25.2) |
|  |  | Latency to touch conspecifics | 485.9 (27.3) | 506.5 (27.4) | 492.5 (19.1) |
|  |  | Total time with conspecifics | 118.1 (20.1) | 80.5 (17.1) | 95.7 (12.8) |
|  |  |  |  |  |  |
