# Supplementary material for: Personality in the cockroach Diploptera punctata: Evidence for stability across developmental stages despite age effects on boldness
Source: PLoS One. 2017 May 10;12(5):e0176564. doi: 10.1371/journal.pone.0176564 (PMC5425029; doi:10.1371/journal.pone.0176564)
Supplement: S1 Appendix — (DOCX) [file pone.0176564.s009.docx]

**Supporting information**

**Sex-specific differential consistency analyses**

On analysing males and females separately, neither sex showed significant consistency in nymph boldness (male: *r_s_* = 0.365, *N* = 10, *P* = 0.300; female: *r_s_* = 0.566, *N* = 12, *P* = 0.055) or exploration (male: *r_s_* = 0.479, *N* = 10, *P* = 0.162; female: *r_s_* = 0.091, *N* = 12, *P* = 0.778). It became apparent that the boldness correlation was unlikely to be weaker for females than for males (S1 fig) and so this justified pooling both sexes for this analysis. In terms of adult boldness, both sexes in fact showed significant differential consistency (male: *r_s_* = 0.583, *N* = 28, *P* = 0.001; female: *r_s_* = 0.555, *N* = 35, *P* = 0.001), justifying pooling these for the main analysis.

**Sex-specific age effects analyses**

When sex-specific Wilcoxon signed-ranks tests were carried out on measures E3 (total time taken), S1 (latency to reach conspecifics) and S3 (total time with conspecifics), the sex differences in magnitude of these responses apparent above (S4 fig) were not significant following a sequential Bonferroni correction for multiple testing. Males appeared less explorative as adults (as they took a longer time to explore all sectors in the exploration assay; *z* = 2.00, *N* = 7, *P* = 0.043) whereas this age effect was not apparent in females (*z* = 38.00, *N* = 12, *P* = 0.285). Females also appeared to be more sociable as adults as evidenced by them spending significantly more time with conspecifics as adults (*z* = 8.00, *N* = 12, *P* = 0.026); males, on the other hand, appeared less sociable as adults as measured by a greater latency to reach conspecifics in the social assay (*z* = 1.00, *N* = 7, *P* = 0.046). Adult females did not appear to show a difference in their latency to reach conspecifics in the social assay (*z* = 52.00, *N* = 12, *P* = 0.091) and adult males did not appear to show a difference in the total time spent with conspecifics (*z* = 18.00, *N* = 7, *P* = 0.116).
